# When Is Evidence Enough Evidence? A Systematic Review and Meta-Analysis of the Trabectome as a Solo Procedure in Patients with Primary Open-Angle Glaucoma

**DOI:** 10.1155/2017/2965725

**Published:** 2017-06-27

**Authors:** Jeffrey T. Y. Chow, Cindy M. L. Hutnik, Karla Solo, Monali S. Malvankar-Mehta

**Affiliations:** ^1^Department of Epidemiology and Biostatistics, Schulich School of Medicine and Dentistry, Western University, London, ON, Canada; ^2^Department of Ophthalmology, Schulich School of Medicine and Dentistry, Western University, London, ON, Canada

## Abstract

The purpose of this systematic review and meta-analysis was to examine the availability of evidence for one of the earliest available minimally invasive glaucoma surgery (MIGS) procedures, the Trabectome. Various databases were searched up to December 20, 2016, for any published studies assessing the use of the Trabectome as a solo procedure in patients with primary open-angle glaucoma (POAG). The standardized mean differences (SMD) were calculated for the change in intraocular pressure (IOP) and number of glaucoma mediations used at 1-month, 6-month, and 12-month follow-up. After screening, three studies and one abstract with analyzable data were included. The meta-analysis showed statistically significant reductions in IOP and number of glaucoma medications used at all time points. Though the Trabectome as a solo procedure appears to lower IOP and reduces the number of glaucoma medications, more high-quality studies are required to make definitive conclusions. The difficulty of obtaining evidence may be one of the many obstacles that limit a full understanding of the potential safety and/or efficacy benefits compared to standard treatments. The time has come for a thoughtful and integrated approach with stakeholders to determine optimal access to care strategies for our patients.

## 1. Introduction

As the second leading cause of blindness in the world, [1] glaucoma is an important disease that affects millions of people. In 2010, there were 60.5 million people in the world living with glaucoma and this number is predicted to rise to 80 million by 2020 [2]. Glaucoma costs the United States economy over 2.9 billion dollars every year from direct costs and productivity losses [3]. The most common type of glaucoma, primary open-angle glaucoma (POAG), occurs when the angle between the cornea and the iris is anatomically open, but functionally impaired, leading to increased pressure in the eye and potential optic nerve damage [4]. There is no cure for POAG. Current treatments are aimed at lowering intraocular pressure (IOP) with the goal of slowing or halting the progression of POAG.

Surgery is typically required when medication and laser treatments fail to deliver the necessary reduction in IOP. Minimally invasive glaucoma surgeries (MIGS) have become more popular due to their perceived safety and lack of complications [5]. One of these MIGS is the Trabectome surgical system developed by NeoMedix Inc. in Tustin, CA.

The Trabectome allows trained ophthalmologists to perform an ab interno partial trabeculectomy, a procedure that uses high-frequency electrocautery to selectively ablate the trabecular meshwork and inner wall of Schlemm's canal. The procedure results in IOP reduction by creating a more direct communication between the anterior chamber and the collector channels [6]. The Trabectome procedure has been reported to reduce the IOP to the midteens and has low complication rates [7]. Potential benefits associated with the Trabectome include being less invasive as an ab interno approach is used and increased compliance as fewer glaucoma medications are needed afterwards [7]. When performed in conjunction with cataract surgery, the benefit to the patient is a single incision that can be used for the combined procedure. Recently, the Trabectome has been used more as a solo procedure, without concurrent cataract surgery. Under the Ontario Health Insurance Plan, the Trabectome is projected to offer a moderate cost savings compared to glaucoma medications over time [8].

In a 2011 review, Vold suggested that more long-term data and randomized controlled trials were required to adequately assess the efficacy of Trabectome [7]. However, no systematic reviews focused on using Trabectome to treat POAG were found in the literature. A recent systematic review of patients with all types of glaucoma found that overall, Trabectome on average reduces the IOP by approximately 31% to a final IOP near 15 mmHg [9]. Because of the multitude and growing number of potential surgical glaucoma treatments available, there is a need to synthesize the literature available for Trabectome to ensure a full understanding of its position in the glaucoma treatment paradigm. While the original objective of the current systematic review was to analyze all data available for the Trabectome and determine its performance as a solo procedure in patients with POAG, a lack of high-quality evidence (despite years of use) resulted in a new objective to discuss the difficulty with obtaining enough evidence to fully understand the benefits of an intervention compared to potential alternatives.

## 2. Methods

### 2.1. Literature Search Strategy

A systematic review was conducted by searching several databases and pertinent grey literature for all relevant articles. PubMed, MEDLINE, EMBASE, Web of Science Core Collection, and CINAHL were searched until December 20, 2016, using a keyword string (see Appendix 1A available online at https://doi.org/10.1155/2017/2965725). Since, there were no MeSH terms or subject headings available for the Trabectome, only keyword searching was used. The search strategy was designed to reflect naming variations for the Trabectome surgical system and the associated surgical procedure. Since the number of results from database searching was low (see Appendix 1B), the concept of POAG was not included in the keywords. Instead, screening questions were used to ensure that only studies with POAG patients were included.

**Figure 1:**
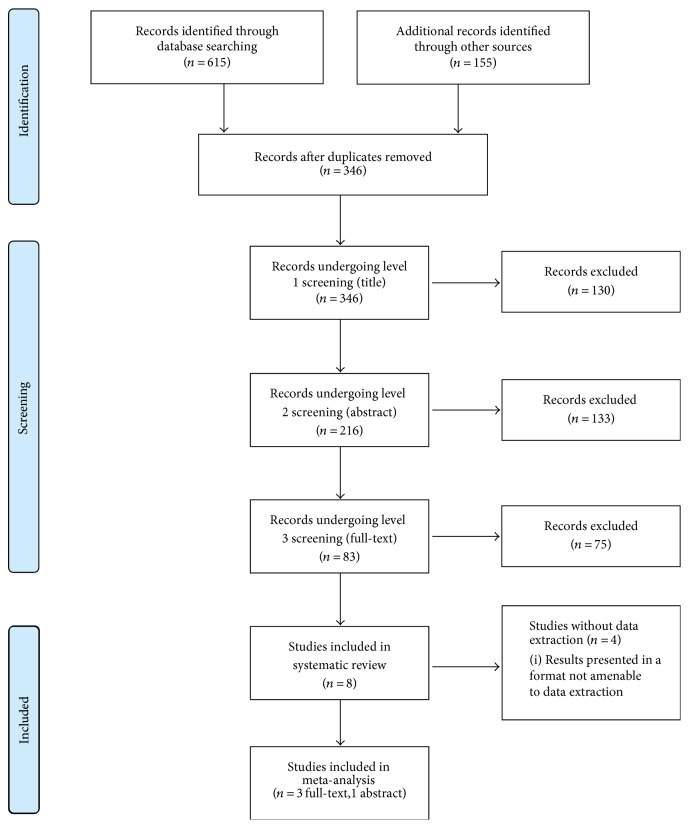
PRISMA flow diagram.

Grey literature was identified by searching ClinicalTrials.gov, the International Clinical Trials Registry Platform, ProQuest Dissertations and Theses, the Networked Digital Library of Theses and Dissertations, the Electronic Thesis Online Service, the Theses Canada Portal, the Canadian Health Research Collection, the Agency for Healthcare Research and Quality, and the Canadian Agency for Drugs and Technologies in Health for all relevant studies. BIOSIS Previews (using the Web of Science platform), the Association for Research in Vision and Ophthalmology (ARVO), the American Academy of Ophthalmology (AAO), and the Canadian Ophthalmological Society (COS) were searched for meeting abstracts that met the criteria described in the database search. Since the Trabectome is manufactured by NeoMedix Inc., its website was also searched for any publications not identified from previous searches. The PRISMA flow diagram for the literature search is presented in Figure 1.

### 2.2. Inclusion and Exclusion Criteria

Primary research studies were included in this systematic review. Secondary research studies such as review articles, case reports, systematic reviews, opinions, and editorials were excluded. Studies presenting outcomes for the Trabectome as a solo procedure in humans with POAG were included. Studies that measured the effect of the Trabectome with concurrent cataract surgery were excluded unless the study also presented the effect of the Trabectome performed alone. No restrictions were made on study location or year of publication. Studies were included if they were published in English and had a sample size over 20. EPPI Reviewer [10] was used to gather data from various published and unpublished sources as well as for duplicate removal. Covidence [11] software was used for the three screening stages, with the titles screened in level 1, the abstracts screened in level 2, and the full texts screened in level 3 (see Appendix 2). Two reviewers (JC and KS) independently screened each study with any disagreements resolved by consensus.

### 2.3. Quality Assessment and Data Extraction

All included studies except for the abstract-only study [12] were assessed for quality using the Downs and Black checklist [13]. All three included full-text studies [14–16] were determined to be of moderate quality with scores of 14, 16, and 15, respectively. Due to limited evidence, none of the studies were excluded from the analysis based on quality. For each of the included studies, the following data was extracted: author name, year of publication, study design, study location, sample size, demographic characteristics of subjects, baseline intraocular pressure, and baseline number of glaucoma medications. Postoperative characteristics such as intraocular pressure and number of glaucoma medications were also extracted for all time points provided in the study.

### 2.4. Statistical Analyses

Meta-analysis was completed using STATA versus 13.0 (STATA Corporation, College Station, TX). Percentage of IOP reduction (IOPR%) and standard error of percentage of IOP reduction (SE_IOPR%_) were calculated using the extracted IOP and standard deviation at each time point according to equations described in similar studies [17, 18]. The outcomes of interest were the standardized mean differences (SMD) for change in intraocular pressure and change in glaucoma medications at 6-month and 12-month follow-up. To calculate the SMD for each study, the difference between the mean pre- and postoperative values for each outcome measure was divided by the SD for that outcomes measure. Each SMD then had weights assigned according to the inverse of its variance in order to compute the average. Based on *I*^2^ statistics and *p* values (>0.01) observed, heterogeneity was determined and fixed-effect or random-effect models were used accordingly. Forest plots were generated for each outcome of interest, and funnel plots were generated to check for publication bias.

## 3. Results

### 3.1. Search Results

Figure 1 describes the flow diagram for the literature search and screening process. The database search located 615 studies (see Appendix 1B), and the grey literature search located a further 155 studies (see Appendix 1C). After removing duplicates, 346 studies were included for screening, with 8 studies remaining after three levels of screening. Four studies had results that were in a format that was not amenable to data extraction (data presented in a graph format or full text not in English) [19–21]. As a result, three studies and one abstract were included in the final meta-analysis. (see Figure 1) [12, 14–16]. Though the full text was in German, the abstract was included since there was sufficient information provided in the abstract [12].

### 3.2. Study Characteristics

All included studies [12, 14–16] measured IOP and glaucoma medications used at baseline and postoperative time points with subgroups for POAG and pseudoexfoliation glaucoma (XFG) patients. The Pahlitzsch et al. study was available as an abstract since the full text was in German [12].

In the Ting et al. study, there were 450 cases of POAG from Canada, Japan, Mexico, and the United States that underwent the Trabectome alone, with an average age of 68 (SD = 15) years, consisting of 40% male [14]. In comparison, the Mizoguchi et al. study included 43 cases of POAG that underwent the Trabectome alone, with an average age of 67.4 (SD = 14.1) years and consisting of 37% male [15]. Akil et al. included 18 cases of POAG from the United States that underwent the Trabectome alone with an average of 72.5 (SD = 7) years and consisting of 44% male [16]. In all four studies where the results were not amenable to replication, Trabectome as a solo procedure reduced the IOP and glaucoma medications used [19–22]. The results for the other four studies are summarized in Table 1 for the POAG cases undergoing the Trabectome surgery.

### 3.3. Publication Bias

**Figure 2:**
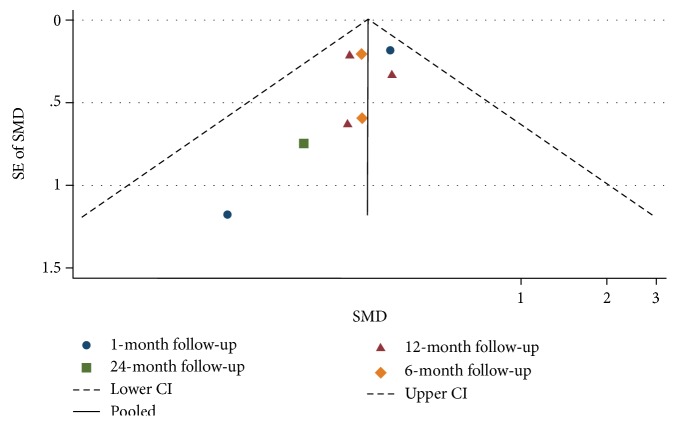
Funnel plot for studies examining change in intraocular pressure (mmHg) by follow-up (months). The dashed line represents the confidence interval (CI).

**Figure 3:**
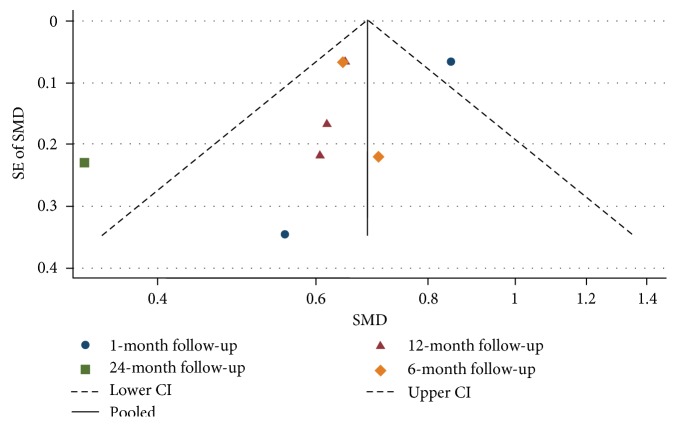
Funnel plot for studies examining change in number of glaucoma medications used by follow-up (months). The dashed line represents the confidence interval (CI).

Visual inspection of the funnel plots presented in Figures 2 and 3 does not show any asymmetry, suggesting a lack of publication bias for the average change in IOP and topical glaucoma medications used, stratified by the length of follow-up.

### 3.4. Effect on Intraocular Pressure

**Figure 4:**
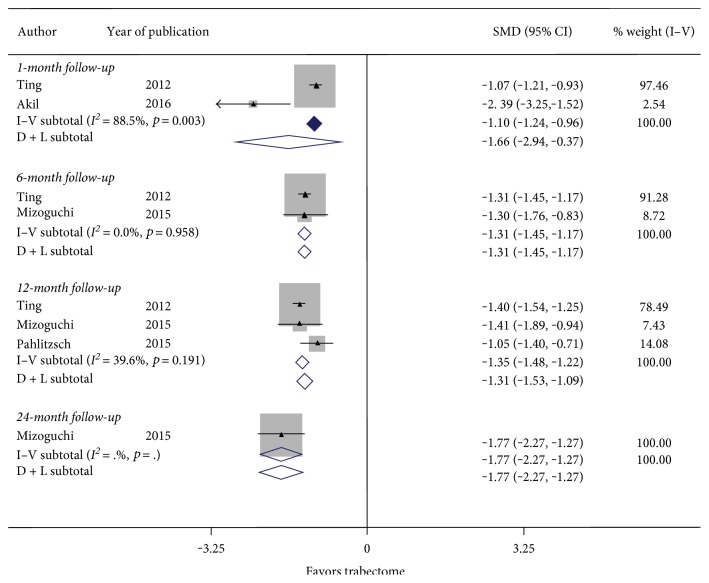
Forest plot for studies examining change in intraocular pressure (mmHg) by follow-up (months).

Figure 4 summarizes the results for the change in IOP at 6-month and 12-month follow-up. There were 2 studies with 1 month follow-up, 2 studies with 6 months follow-up, and 3 studies with 12 months follow-up. There was significant heterogeneity between studies examining 1 month follow-up (*I*^2^ = 88.5%, *p* value = 0.03), but nonsignificant heterogeneity between studies examining 6 months follow-up (*I*^2^ = 0.0%, *p* value = 0.958) and 12 months follow-up (*I*^2^ = 39.6%, *p* value = 0.191). Figure 4 showed a nonstatistically significant reduction in IOP with a SMD of −1.66 (CI: [−2.94, −0.37]) at 1 month and a statistically significant reduction in IOP with a SMD of −1.31 (CI: [−1.45, −1.17]) at 6 months and −1.35 (CI: [−1.48, −1.22]) at 12 months follow-up. This suggests that the significant reduction in IOP from the Trabectome procedure persists even after 12 months.

### 3.5. Effect on Glaucoma Medication Use

**Figure 5:**
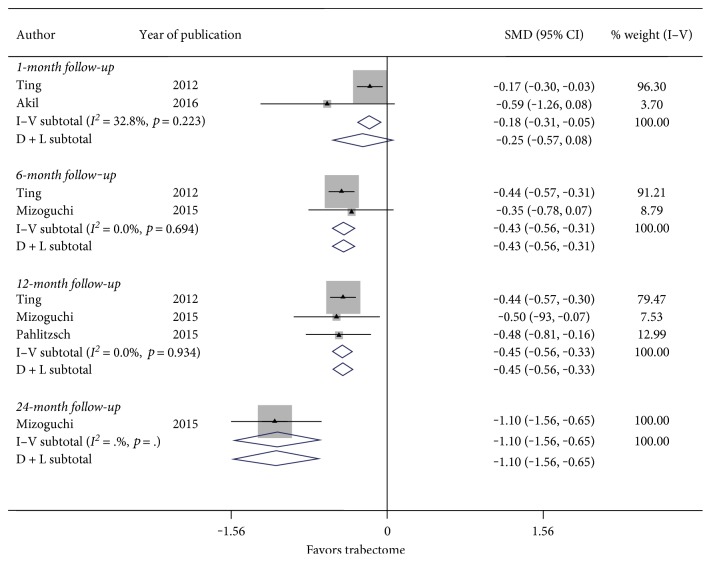
Forest plot for studies examining change in number of glaucoma medications used by follow-up (months).

Figure 5 summarizes the results for the change in glaucoma medications used at 6 months and 12 months follow-up. There were 2 studies considering follow-up of 6 months and 3 studies inspecting 12 months follow-up. Nonsignificant heterogeneity between studies examining follow-up at 1 month (*I*^2^ = 32.8%, *p* value = 0.223), 6 months (*I*^2^ = 0.0%, *p* value = 0.694), and 12 months (*I*^2^ = 0.0%, *p* value = 0.934) was used to determine the fixed-effect computations. In Figure 5, there was a statistically significant reduction in postoperative glaucoma medications used with a SMD of −0.18 (CI: [−0.31, −0.05]) at 1 month, SMD of −0.43 (CI: [−0.56, −0.31]) at 6 months, and a SMD of −0.45 (CI: [−0.56, −0.33]) at 12 months follow-up. Thus, Trabectome surgery may significantly reduce dependence on glaucoma medications at 12 months follow-up.

## 4. Discussion

A systematic review of the literature was conducted to determine the performance of the Trabectome as a solo procedure in patients with POAG. The primary outcomes measured were IOP and glaucoma medications used. Various bibliographic databases and grey literature were searched resulting in inclusion of three relevant full-text studies and one abstract after three levels of screening, indicating a lack of published evidence available for this topic. Thus, it would be ideal if more studies could be conducted to better understand the optimal role of Trabectome in IOP management and topical glaucoma medication management.

In all included studies, the results suggested that the Trabectome surgery resulted in significant reduction in IOP and glaucoma medications in POAG patients [12, 14–16]. While the included studies showed a 25–34% reduction in IOP at 12 months follow-up and the SE_IOPR%_ ranged from 33–35%—large standard errors suggest that the Trabectome may have differing levels of efficacy between patients. In addition, the Trabectome was not compared to a placebo (or standard of care/alternative treatment) as the key objective of this study was to determine whether the primary diagnosis of the patients had an effect on the Trabectome's success. Without an adequate control group, a strong conclusion cannot be made for the efficacy of the Trabectome as a solo procedure in POAG patients.

A recent systematic review that included all glaucoma subjects, regardless of type, also found that IOP and glaucoma medications used decreased significantly from baseline [9]. A significant strength of this analysis stems from the fact that all the included studies had coherent results of reduction in IOP as well topical glaucoma medications due to Trabectome surgery.

The study limitations for meta-analyses such as this are necessary to ponder before inferences may be considered. The primary limitation of this systematic review was the narrow inclusion criteria and the resulting low number of included studies. One reason for the low number of included studies was the lack of availability of studies that specifically examined the effect of the Trabectome in patients with POAG. In two of the included studies, the XFG group had a higher mean reduction in IOP than the POAG group, which could be due to the higher preoperative IOP levels in XFG patients [14, 19]. The Mizoguchi et al. study also showed that the XFG had a higher percent mean reduction in IOP than the POAG group, but the preoperative IOP levels were higher in the POAG patients [15]. This difference between primary and secondary glaucoma reinforces the decision to exclude studies where the POAG results are not presented separately from the secondary open-angle glaucoma such as XFG. Many studies located in the literature search were excluded because they presented the results for all types of glaucoma and pooled patients with differing glaucoma diagnoses. The lack of evidence stratified by glaucoma subgroup makes clinical decision-making difficult since physicians will be unable to determine whether the intervention is suitable for their specific patients. Further, complication and failure rates may have more to do with the type of glaucoma rather than the procedure.

Secondly, it is necessary to consider the quality of the included studies. In this meta-analysis, the Downs and Black checklist [13] was employed and none of the included studies were found to be of high quality. The included studies lacked elements such as randomization, blinding, and a control group. Nevertheless, due to limited number of studies available for the analysis, all were included, irrespective of their quality. This is a recognized, but necessary, limitation due to the few clinical studies currently available.

Thirdly, meta-analysis of observational studies is influenced by inherent biases in the included articles [23]. For example, a multitude of other factors such as level of education, ethnicity, income status, socioeconomic status, previous ocular and nonocular surgeries, family history, other ocular and nonocular diseases, preoperative and postoperative medications, number of medications, and comorbidities (e.g., high blood pressure, diabetes, stroke, and heart conditions) could influence the estimates in the original studies. Potential bias related to industry sponsorship of a study also exists, as well as methods of patient selection. Variations in surgical technique may be a major factor as well.

The results of this meta-analysis showed reduction in IOP and topical glaucoma medications after Trabectome surgery. However, the current literature suggests that additional research is warranted to best understand how to maximize the utility of Trabectome in the management of glaucoma patients. Even though the Trabectome has been available for over ten years, no randomized controlled trials have been published comparing the Trabectome with other potential glaucoma treatments [24]. As a result, it is challenging for hospitals, physicians, and other decision-makers to determine if the available evidence is sufficient to warrant publicly funded access to innovations in technology such as the Trabectome. A notable lack of published research on rates of early and late postoperative complications suggests that more research is also needed in this area.

## 5. Conclusions

In conclusion, this systematic review suggests that the Trabectome may be helpful in reducing the IOP and the number of glaucoma medications used in POAG patients. However, there is a need for sufficient evidence to determine Trabectome's effectiveness as a solo procedure in treating POAG due to the low number and quality of available studies. These results are concerning as the Trabectome has been used to treat open-angle glaucoma since 2006 in the United States without sufficient evidence to support anecdotal experience. The Trabectome is just one example of a technological innovation in which funding challenges have impeded access to the evidence that would allow a more definitive understanding of its role in the glaucoma treatment paradigm. There is a strong need for more studies to be conducted on the Trabectome to show effectiveness, especially randomized controlled trials and trials comparing the Trabectome to control treatments. As the Trabectome is not the only intervention in which there is a paucity of relevant research to allow evidence-based decision-making, it may be time for all stakeholders (physicians, hospitals, government, and industry) to come together to determine collective strategies that will ensure access to optimal care for patients with glaucoma.

## Supplementary Material

Appendix 1A: Keyword String; Appendix 1B: Database Search; Appendix 1C: Grey Literature Search; Appendix 2: Screening Questions.

## Figures and Tables

**Table 1:** Reported pre- and postoperative IOP and number of glaucoma medications for POAG cases undergoing Trabectome as a solo procedure.

Author, year	Time point	*N*	Mean IOP (mmHg (SD))	IOPR%	SE_IOPR%_	*N*	Mean number of medications (mean (SD))	Mean reduction in number of medications
Ting et al. [14]	Baseline	450	25.5 (7.9)	—	—	450	2.73 (1.33)	—
	1 day	450	16.5 (7.9)	35%	44%	450	2.21 (1.73)	0.52
	1 month	420	18.1 (5.8)	29%	38%	420	2.50 (1.45)	0.23
	3 months	384	17.6 (5.3)	31%	37%	384	2.34 (1.42)	0.39
	6 months	327	17.3 (4.0)	32%	35%	327	2.14 (1.34)	0.59
	12 months	293	16.8 (3.9)	34%	35%	293	2.16 (1.29)	0.57

Mizoguchi et al. [15]	Baseline	82	23.5 (7.2)	—	—	43	2.8 (0.8)	—
	6 months	74	16.2 (3.4)	31%	34%	37	2.5 (0.9)	0.3
	12 months	60	15.7 (3.0)	33%	33%	29	2.4 (0.8)	0.4
	18 months	43	15.3 (2.4)	35%	32%	23	2.5 (0.7)	0.3
	24 months	22	14.1 (2.2)	40%	32%	8	1.8 (1.0)	1

Pahlitzsch et al. [12]	Baseline	—	19.8 (5.9)	—	—	—	Not available	—
	12 months	—	14.8 (3.2)	25%	34%	—	2.1 (1.2)	—

Akil et al. [16]	Baseline	18	24.2 (4.7)	—	—	18	2.6 (1.2)	—
	1 month	18	14.6 (3.2)	40%	23%	18	1.7 (1.2)	0.9

IOP: intraocular pressure; IOPR%: percentage reduction in intraocular pressure; SE_IOPR%_: standard error of percentage reduction in intraocular pressure. IOP refers to intraocular pressure and SD refers to standard deviation.
