# Supplementary material for: When Is Evidence Enough Evidence? A Systematic Review and Meta-Analysis of the Trabectome as a Solo Procedure in Patients with Primary Open-Angle Glaucoma
Source: J Ophthalmol. 2017 Jun 27;2017:2965725. doi: 10.1155/2017/2965725 (PMC5504928; doi:10.1155/2017/2965725)
Supplement: Supplementary file 1 — Appendix 1A: Keyword String; Appendix 1B: Database Search; Appendix 1C: Grey Literature Search; Appendix 2: Screening Questions. [file 2965725.f1.pdf]

Appendix 1A – Keyword String

**Keyword string used**
trabectome OR ab interno trabeculotom* OR ab interno trabeculectom* OR trabeculotom* ab interno OR trabeculectom* ab interno

Appendix 1B – Database Search

**Database Search**					
PubMed	Medline	Embase	Web of Science	CINAHL	Total
161	133	146	160	15	615

Appendix 1C – Grey Literature Search

**Grey Literature Search**	
	**Number Found**
ClinicalTrials.gov, the International Clinical Trials Registry Platform, ProQuest Dissertations and Theses, the Networked Digital Library of Theses and Dissertations, the Electronic Thesis Online Service, the Theses Canada Portal, the Canadian Health Research Collection, the Agency for Healthcare Research and Quality, and the Canadian Agency for Drugs and Technologies in Health	0
BIOSIS Previews (using the Web of Science platform	75
Association for Research in Vision and Ophthalmology (ARVO) meeting abstracts	45
American Academy of Ophthalmology (AAO) meeting abstracts	26
Canadian Ophthalmological Society (COS) meeting abstracts	3
NeoMedix website	6
Total number of grey literature located	155

Appendix 2 – Screening Questions

For all screening questions, answer options were Yes, No, or Unclear.

Level 1 Screening (kappa= 0.70)

1. Does this study not occur in non-humans or cadavers? (Indicate Yes if no mention of species of subjects)

2. Is this a primary research study (i.e. not an editorial, opinion, review article, systematic review, comments, replies, discussion, and erratum)? (Indicate Unclear if no mention of study type)

3. Is the disease of interest open angle glaucoma? (Indicate Unclear if glaucoma is mentioned without the subtype specified)

4. Does this study look at the Trabectome device? (Indicate Unclear if technique not mentioned or general techniques such as ab interno trabeculectomy)

Level 2 Screening (kappa= 0.74)

1. Is the study looking at the Trabectome device in patients with Primary Open Angle Glaucoma? (Indicate Unclear if type of glaucoma is not specified; indicate No if secondary open angle glaucoma is specified without primary open angle glaucoma patients analyzed separately)

2. Is this a primary research study with sample size over 20? (i.e. not an editorial, opinion, review article, systematic review)

Level 3 Screening (kappa= 0.48)

1. Are there results for the Trabectome performed alone (without concurrent cataract surgery/ phacoemulsifcation)?

2. Are the outcomes change in IOP or change in number of glaucoma medications included?
